# Recent Development of Nanocomposite Membranes for Water and Wastewater Treatment

**DOI:** 10.3390/nano13101686

**Published:** 2023-05-20

**Authors:** Ahmad Fauzi Ismail, Pei Sean Goh, Norhaniza Yusof

**Affiliations:** Advanced Membrane Technology Research Centre, Universiti Teknologi Malaysia, Skudai 81310, Malaysia

The field of membrane technology has experienced significant growth in recent years, especially in the areas of wastewater treatment and desalination. The increasing need for clean water has driven the development of high-performance membranes that are versatile, durable, and multifunctional. Nanomaterials have emerged as a promising tool that offers the flexibility to tailor their physical and chemical properties in order to meet specific needs. This has led to the development of nanocomposite membranes that hold great potential in the field of water and waste treatment. This Special Issue, entitled ‘Recent Development of Nanocomposite Membranes for Water and Wastewater treatment’, has brought together research articles and reviews from leading experts in this field that discuss topics such as the synthesis of nanomaterials for membrane fabrication as well as the modification and functionalization of nanocomposite membranes.

In total, five articles were published in this Special Issue, including four research articles and one review article. Saleem et al. addressed the challenges of the forward osmosis process for desalination through membrane design. Thin-film composite membranes (TFC) were fabricated with polyethersulfone produced from a solution blown-spun nanofiber support and a selective layer incorporated with graphene quantum dots derived from banyan tree leaves [1]. The optimized membrane exhibited an improved performance in terms of water flux, salt rejection, and chlorine resistance compared to the neat TFC membranes. The authors also reported the preparation of a forward osmosis desalination membrane incorporated with graphene quantum dots derived from eucalyptus tree leaves [2]. Wan Ikhsan et al. presented a one-pot synthesis of a nanocomposite coating with hydrophilic and oleophobic properties using the sol–gel technique [3]. The coating was designed to improve the separation of oil-in-water emulsion in polymer membranes. Philipp et al. investigated the effectiveness of using ultrafiltration and reverse osmosis in combination for the purification and recycling of slaughterhouse wastewater [4]. Lab- and bench-scale experiments were performed, in which the hybrid system achieved a reduction of more than 98% for the total organic carbon and chemical oxygen demand. Sarbatly and Chiam presented a review on the recent developments in nanofiber membranes used for oily wastewater treatment [5]. The review covers various production techniques and modifications implemented to improve the separation efficiency. The highly interconnected pore structure and modified wettability of these membranes can improve water flux and reduce fouling.

This Special Issue presents a collection of articles that cover a wide range of topics related to membrane technology, including new materials, process designs, and applications in different industries. Interdisciplinary research on material chemistry and separation engineering applications is critical for enhancing the performance of nanocomposite membranes. The contributions to this Special Issue will enable readers to gain insight into the latest research developments in this area and highlight the significance of nanocomposite membranes for addressing the challenges of water and wastewater treatment. 
